# Escape is a more common mechanism than avidity reduction for evasion of CD8+ T cell responses in primary human immunodeficiency virus type 1 infection

**DOI:** 10.1186/1742-4690-8-41

**Published:** 2011-06-02

**Authors:** Emma L Turnbull, Joshua Baalwa, Karen E Conrod, Shuyi Wang, Xiping Wei, MaiLee Wong, Joanna Turner, Pierre Pellegrino, Ian Williams, George M Shaw, Persephone Borrow

**Affiliations:** 1 Nuffield Department of Clinical Medicine, University of Oxford, Weatherall Institute of Molecular Medicine, John Radcliffe Hospital, Headington, Oxford, OX3 9DS, UK; 2 Departments of Medicine and Microbiology, University of Alabama at Birmingham, 720 South 20th Street, KAUL 816, Birmingham, AL 35294-0024, USA; 3 Centre for Sexual Health & HIV Research, Mortimer Market Centre, London, WC1E 6AU, UK

## Abstract

**Background:**

CD8+ T cells play an important role in control of viral replication during acute and early human immunodeficiency virus type 1 (HIV-1) infection, contributing to containment of the acute viral burst and establishment of the prognostically-important persisting viral load. Understanding mechanisms that impair CD8+ T cell-mediated control of HIV replication in primary infection is thus of importance. This study addressed the relative extent to which HIV-specific T cell responses are impacted by viral mutational escape versus reduction in response avidity during the first year of infection.

**Results:**

18 patients presenting with symptomatic primary HIV-1 infection, most of whom subsequently established moderate-high persisting viral loads, were studied. HIV-specific T cell responses were mapped in each individual and responses to a subset of optimally-defined CD8+ T cell epitopes were followed from acute infection onwards to determine whether they were escaped or declined in avidity over time. During the first year of infection, sequence variation occurred in/around 26/33 epitopes studied (79%). In 82% of cases of intra-epitopic sequence variation, the mutation was confirmed to confer escape, although T cell responses were subsequently expanded to variant sequences in some cases. In contrast, < 10% of responses to index sequence epitopes declined in functional avidity over the same time-frame, and a similar proportion of responses actually exhibited an increase in functional avidity during this period.

**Conclusions:**

Escape appears to constitute a much more important means of viral evasion of CD8+ T cell responses in acute and early HIV infection than decline in functional avidity of epitope-specific T cells. These findings support the design of vaccines to elicit T cell responses that are difficult for the virus to escape.

## Background

Virus-specific CD8+ T cell responses are expanded as the acute burst of viral replication occurs in primary HIV infection [1-3] and are thought to make an important contribution to resolution of acute viraemia and establishment and maintenance of the level of ongoing virus replication [4-6]. Understanding of mechanisms that may undermine the ability of HIV-specific CD8+ T cell responses to achieve and sustain good control of virus replication in the critical initial phase of infection is of importance to inform the rational design of prophylactic and therapeutic strategies targeting cell-mediated responses to induce optimal containment of HIV infection. Mechanisms proposed to contribute to impairment of T cell-mediated control of viral replication during acute/early infection include virus mutational escape from CD8+ T cell responses [4,7], reduction in the functional avidity of CD8+ T cell responses (possibly due to the exhaustion and deletion of higher avidity T cell clones [8,9]) and acquisition of defects in the functional capacity of HIV-specific T cells [10-14]. However, the relative contribution of each of these mechanisms to impairment of HIV control during acute/early infection is not well understood.

HIV evolution to acquire mutations conferring partial or complete escape from epitope-specific CD8+ T cell responses occurs commonly at the population level [15,16] and has been shown to take place during both acute/early infection [3,4,6,7,17] and chronic infection [18,19]. Evolution of mutations in or around T cell epitopes can promote escape via mechanisms including impaired antigen processing of the epitope, altered binding of the epitope to the cognate human leukocyte antigen (HLA) class I molecule and altered interaction of the HLA class I-peptide complex with the T cell receptor (TCR). The impact of escape from any given epitope-specific T cell response will depend on the relative contribution of that response to overall containment of virus replication and the fitness cost associated with viral sequence variation. In some cases, escape from the T cell response to a single epitope can lead to loss of control of virus replication and disease progression [18,19].

The functional avidity of T cell responses has been shown to influence their efficacy in both viral and tumour models [20-22]. Higher avidity T cell responses tend to be more efficacious for controlling virus replication because they are sensitive to lower antigen concentrations and preferentially activated early in infection when antigen is limiting, and they initiate target cell lysis better than lower avidity T cells at any given antigen density [23]. *In vitro *studies also suggest that HIV-specific CD8+ T cells must exceed an epitope-dependent avidity threshold in order to mediate lysis of infected cells, suggesting that small differences in avidity can have a very marked effect on antiviral efficacy [24]. A recent study also reported a relationship between T cell avidity and polyfunctionality, finding that high avidity HIV-specific T cells are typically polyfunctional and capable of mediating potent suppression of viral replication *in vitro *[25]. However, higher avidity clones are also more prone to becoming exhausted and deleted from the repertoire [8,9], and their loss may be associated with reduced control of virus replication. Maintenance of high avidity clones may correlate with more favourable disease prognosis [9].

In this study, we addressed the relative frequency with which mutational escape and reduction in T cell response avidity occurred in acute and early HIV infection, to gain insight into the potential impact of these two mechanisms on T cell-mediated containment of virus replication at this time. Sequence variation and escape were found to occur much more frequently than reduction in T cell avidity during the first year of infection.

## Results

### Identification of CD8+ T cell responses in subjects acutely infected with HIV

18 patients presenting with symptomatic primary HIV-1 infection who were sampled at sequential time-points from acute infection onwards were studied (Table 1). The first sampling time-point was at a mean of 20 days following onset of symptoms (DFOSx) (median = 18.5 DFOSx, range = 5-55 DFOSx), when the mean viral load was 577,594 copies/ml plasma (range 1,200 - 4,337,100 copies/ml) and the majority of subjects had only recently begun to seroconvert. After the acute phase of infection, the majority of subjects controlled virus replication relatively poorly, with only 3 patients containing virus replication to below 2,000 HIV RNA copies/ml (Table 1).

**Table 1:** Clinical and sampling profiles of patients studied.

Patient	HLA class I type			Time of last HIV Ab negative test (DFOSx^1^)	Time of first fully positive HIV Ab test (DFOSx)	First sampling time-point studied (DFOSx)	Viral load at first PBMC sampling time point (RNA copies/ml plasma)	Setpoint persisting viral load established after the acute phase of infection (RNA copies/ml plasma)
MM7	A*02 A*03	B*07 B*44	Cw*05 Cw*07	Evolving at 11	16	23	690,000	128,925

MM9	A*01 A*66	B*41 B*08	Cw*07 Cw*07	7	19	19	142,700	19,379

MM12	A*03 A*68	B*07 B*44	Cw*07 Cw*07	ND^2^	7	16	1,555,700	97,970

MM13	A*01 A*01	B*08 B*57	Cw*06 Cw*07	ND	15	16	131,800	15,348

MM26	A*02 A*68	B*51 B*35	Cw)15 Cw*04	Evolving at 37	49	55	56,200	34,493

MM27	A*02 A*03	B*07 B*44	Cw*05 Cw*07	Evolving at 12	26	28	353,200	48,360

MM28	A*11 A*30	B*13 B*35	Cw*04 Cw*06	6	9	6	4,337,100	12,322

MM33	A*02 A*68	B*07 B*44	Cw*05 Cw*07	Evolving at 9	12	12	1,451,400	73,958

MM34	A*01 A*24	B*51 B*35	Cw*12 Cw*12	Evolving at 10	17	17	29,900	8,522

MM39	A*02 A*03	B*15 B*35	Cw*03 Cw*04	Evolving at 3	23	5	350,600	8,546

MM43	A*02 A*02	B*55 B*40	Cw*10 Cw*09	Evolving at 6	13	21	Not available (898,100 at 27 DFOSx)	64,565

MM45	A*03 A*03	B*07 B*51	Cw*07 Cw*15	Evolving at 1	22	22	23,200	1,917

MM46	A*02 A*11	B*08 B*52	Cw*07 Cw*12	1	5	5	224,100	81,011

MM47	A*24 A*24	B*39 B*65	Cw*02 Cw*07	Evolving at -1	8	28	17,000	15,839

MM48	A*24 A*26	B*62 B*27	Cw*01 Cw*09	1	Evolving at 16	22	40,100	4,266

MM51	A*02 A*30	B*13 B*44	Cw*05 Cw*06	Evolving at 5	39	18	39,400	26,557

MM55	A*01 A*33	B*14 B*15	Cw*07 Cw*08	6	24	31	1,200	50

MM56	A*02 A*24	B*35 B*57	Cw*04 Cw*06	4	ND	14	15,000	1,023

^1^DFOSx = days following onset of symptoms; ^2^ND = not determined

In each individual, we mapped the specificity of the primary HIV-specific T cell response using an interferon (IFN)γ enzyme-linked immunosorbent spot (ELISPOT) matrix-based peptide screening approach. Patient peripheral blood mononuclear cells (PBMC) pooled from time-points within the first six months of infection (typically from 4-6 months FOSx) were tested for reactivity to overlapping peptides spanning either the clade B consensus (2001) sequence or (in four subjects) the patient's autologous virus sequence determined at the earliest available sampling time-point. The HIV-specific T cell response at the time of mapping targeted a mean of 8.2 epitopic regions (range = 2-17 epitopic regions) and the three most frequently recognised proteins were Gag, Nef and Pol, accounting for 24%, 22% and 22% of all epitopic regions detected, respectively.

T responses to different viral epitopes expand asynchronously in primary HIV infection [3]: typically, rapid expansion of responses to just a limited number of epitopes is initially observed, followed by successive waves of expansion and contraction of responses to other epitopes so that the overall response breadth increases over time, with multiple shifts occurring in the pattern of epitope immunodominance. Having mapped the epitopes recognised at ~4-6 months FOSx in each patient, we then performed a kinetic analysis of the magnitude of the response to each epitopic region during acute/early infection so that a subset of responses appropriate for further study could be selected. Responses chosen were those that were present at a magnitude high enough to permit characterisation at the earliest sampling time-point during acute infection and remained of sufficient magnitude for study over the first year of infection, and where the optimal CD8+ T cell epitope sequence within the epitopic region could readily be identified. In total, we analysed 33 T cell responses to HIV-1 epitopes of 24 different specificities (1-3 epitopes/patient), located in diverse HIV-1 proteins and restricted by a range of HLA class I alleles (Table 2). The responses studied included some that were immunodominant and others that were sub-dominant in the individual's acute/early HIV-specific T cell response. 16 of 33 epitopes (48%) studied were contained within Nef.

**Table 2:** Longitudinal autologous epitope sequence data.

Patient	Clade B consensus epitope sequence(s)	**Autologous epitope sequence(s)**^**1**^					
MM7	HLA-A3 RLRPGGKKK (Gag p17 _20-28_)	**d23** RLRPGGKKK	**d87** RLRPGGKKK	**d553** RLRPGGKKK	**d766** RLRPGGKK***R***	**d934** RLRPGGKK***R***	
	HLA-A3 QVPLRPMTYK (Nef _73-82_)	QVPLRPMTYK	QVPLRPM*T*/***N***YK^2^	QVPLRPM*T*/***N***YK	QVP*L/**V***RPM*T*/***N***YK	QVP*L*/***VG***PMTYK	

MM9	HLA-B8 FLKEKGGL (Nef _90-97_)	**d26** FLKEKGGL	**d54** FLKEKGGL	**d105** FLKEKGGL	**d273** FLKEKGGL	**d343** FLKEKGGL	
	HLA-Cw07 KRQDILDLWVY (Nef _105-115_)	KRQDILDLWVY	KRQ*D/**E***ILDLWVY	KRQ*D/**E***ILDLWVY	***R***RQ***E***ILDLWVY	***R***RQ***E***ILDLWVY	

MM12	HLA-A3 QVPLRPMTYK (Nef _73-82_)	**d16** QVPLRPMTYK	**d40** QVPLRPMTYK	**d139** QVPLRPMTYK	**d230** QVPLRPMTYK	**d321** QVPLRPMTYK	**d487** QVPLRPMTYK
	HLA-A3 QIYAGIKVK (RT _269-277_)	QIYAGIKVK	QIYAGIKVK	QIYAGIKV***R***	QIYAGIKV***R***	QIYAGIKV***R***	QIYAGIKV***R***

MM13	HLA-B8 FLKEKGGL (Nef _90-97_)	**d16** FLKEKGGL	**d45** FLKEKGGL	**d96** FLKE*K/**E***GGL	**d275** FLKE***E***GGL	**d544** FLKE***E***GGL	
	HLA-B57 KAFSPEVIPMF (Gag p24 _30-40_)	KAFSPEVIPMF	KAFSPEVIPMF	KAFSPEVIPMF	KAFSPEVIPMF	KAFSPEVIPMF	
	HLA-B57 HTQGYFPDWQ (Nef _116-125_)	HTQGYFPDWQ	HTQGYFPDWQ	HTQGYFPDWQ	HTQGYFPDWQ	HTQGYFPDWQ	

MM26	HLA-B7 KPQVPLRPMTY (Nef _71-81_)	**d55** KPQVPLRPMTY	**d169** ***R***PQVPLRPMTY	**d253** ***R***PQVPLRPMTY	**d415** ***R***PQVPLRPMTY		
	HLA-A2 YTAFTIPSI (RT _127-135_)	YTAFTIPSI	YTAFTIPS*I/**T***	YTAFTIPS***T***	YTAFTIPS***T***		

MM27	HLA-A2 YTAFTIPSI (RT _127-135_)	**d28** YTAFTIPSV	**d53** YTAFTIPSV	**d81** YTAFTIPSV	**d299** YTAFTIPS*V/**I***	**d466** YTAFTIPS***I***	

MM28	HLA-A11 AAVDLSHFLK (Nef _83-92_)	**d9** AAVDLSHFLK	**d34** AA***L***DLSHFLK	**d198** AA***L***DLSHFLK	**d405** ***G***A***L***DLSHFLK		

MM33	HLA-B44 EEMNLPGRW (Protease _34-42_)	**d12** EEMNLPGRW	**d96** E***D***MNLPGRW	**d201** E***D***MNLPGRW	**d391** E***D***MNLPGRW		

MM34	HLA-B35 DPNPQEVVL (Gp160 _78-86_)	**d17** DPNPQEVVL	**d45** DPNPQEVVL	**d192** DP*N*/***S***PQEVVL	**d353** DP*N/**S***PQEVVL		
	HLA-A24 RYPLTFGWCF (Nef _134-143_)	RYPLTFGWCF	RYPLTFGWCF	R***F***PLTFGWCF	R***F***PLTFGWCF		

MM39	HLA-A3 RLRPGGKKK (Gag p17 _20-28_)	**d11** RLRPGGKKK	**d92** RLRPGGKKK	**d179** RLRPGGKKK	**d358** RLRPGGKKK		
	HLA-A3 QVPLRPMTYK (Nef _73-82_)	QVPLRPMTYK	QVPLRPMTYK	QVPLRPMTYK	QVPLRPMTYK		

MM43	HLA-B40 KEKGGLEGL (Nef _92-100_)	**d21** KEKGGLEGL	**d101** KEKGGLEGL	**d228** KEKGGLEGL	**d368** KEKGGLEGL		
	HLA-A2 ALQDSGLEV (RT _485-493_)	ALQDSGLEV	ALQDSGLEV	ALQDSGLEV	ALQDSGLEV		
	HLA-A2 LEWRFDITL (Nef _181-189_)	LEWRFDITL	LEWRFDITL	L*E**/Q/P/A***WRFDITL	L***A***WRFDITL		

MM45	HLA-A3 RLRPGGKKK (Gag p17 _20-28_)	**d22** RLRPGGKKK	**d87** RLRPGGKKK	**d213** RLRPGGKKK			

MM46	HLA-A2 LVWKFDSRL (Nef _181-189_)	**d5** LVWKFDSRL	**d56** LVWKFDSRL	**d175** LVWKFDSRL	**d530** LVWKFDSRL		
	HLA-A11 RLAFHHVAR (Nef _188-196_)	RLAFHHVAR	RLAFHHVAR	RLAFHH***A***AR	RLAFHH***A***AR		

MM47	HLA-B14 ERYLKDQQL (Gp160 _584-592_)	**d28** ERYLKDQQL	**d57** ERYLKDQQL	**d84** ERYLQDQQL	**d113** ERYLKDQQL	**d217** ERYL***Q***DQQL	**d402** ERYL***Q***DQQL
	HLA-A24 RYPLTFGWCY (Nef _134-143_)	RYPLTFGWCY	R***F***PLTFGWCY	R***F***PLTFGWCY	R***F***PLTFGWCY	R***F***PLTFGWCY	R***F***PLTFGWCY

MM48	HLA-B27 KRWIIMGLNK (Gag p24 _131-140_)	**d22** KRWIIMGLNK	**d50** KRWIIMGLNK	**d113** KRWIIMGLNK	**d204** KRWII*M/**L***GLNK		
	HLA-A24 RYPLTFGWCF (Nef _134-143_)	RYPLTFGWCF	RYPLTFGWCF	RYPLTFGWCF	RYPLTFGWCF		

MM51	HLA-B13 RQANFLGKI (Gag p2p7p1p6 _66-74_)	**d18** RQANFLGKI	**d86** RQANFLGKI	**d207** RQANFLGKI	**d389** RQANFLGKI		

MM55	HLA-B14 DRFYKTLRAEQ (Gag p24 _166-176_)	**d31** DRFYKTLRAEQ	**d94** DRFYKTLRAEQ	**d227** DRFYKTLRAEQ	**d347** DRFYKTLRAEQ		
	HLA-B14 ERYLKDQQL (Gp160 _584-592_)	ERYLKDQQL	ERYLKDQQL	ERYLKDQQL	ERYLKDQQL		
	Unknown RDISGWILSTY (Rev _53-63_)	RDISGWILSTY	RDISGWILSTY	RDISGWILSTY	RDISGWILS***T/A***Y		

MM56	HLA-B57 TSTLQEQIGW (Gag p24 _108-117_)	**d14** TSTLQEQIGW	**d75** TSTLQEQIGW	**d186** TS***N***LQEQIGW	**d375** TS***N***LQEQIGW		

^1 ^The autologous virus sequence of the epitope at the indicated timepoint (day (d) FOSx is shown. Areas of amino acid variation within the epitope are indicated in bold italics and underlined.

### Intra-epitopic sequence variation is common during the first year following presentation with HIV-1 infection

To address the extent to which T cell responses may have been escaped by viral mutation, autologous virus population sequencing of epitope-containing regions was performed at selected time-points over at least the first year following presentation (with the exception of patients MM45 and MM48 whose last available sequence information was at 213 and 204 DFOSx respectively) (Table 2). One or more sites of amino acid variation were observed during year 1 in or around 26/33 (79%) of the epitopes studied. Of these, 17/33 (52%) showed only intra-epitopic sequence variation (Table 2), 2/33 (6%) showed changes both within the epitope (Table 2) and in the flanking regions and 7/33 (21%) exhibited variation in the epitope flanking regions only. In 14/19 (74%) of the cases of intra-epitopic sequence variation, the changes became fixed in the virus population within the first year. Most were limited to a single residue; however, in two cases fixation of substitutions at two sites occurred. Some mutations arose very rapidly: in patients MM28 and MM47, mutations were fixed in the viral population by 34 and 57 DFOSx respectively. 6/19 (32%) had varied within 3 months FOSx, and more than three-quarters (15/19, 79%) had varied by 6 months FOSx. For the 9 epitopes exhibiting amino acid variation within the flanking regions, changes were typically observed at 1 or 2 sites; and the majority became fixed within 1 year. Mutations were observed in or around at least one of the subset of epitopes sequenced in 17 of the 18 patients included in the study.

**Figure 1:**
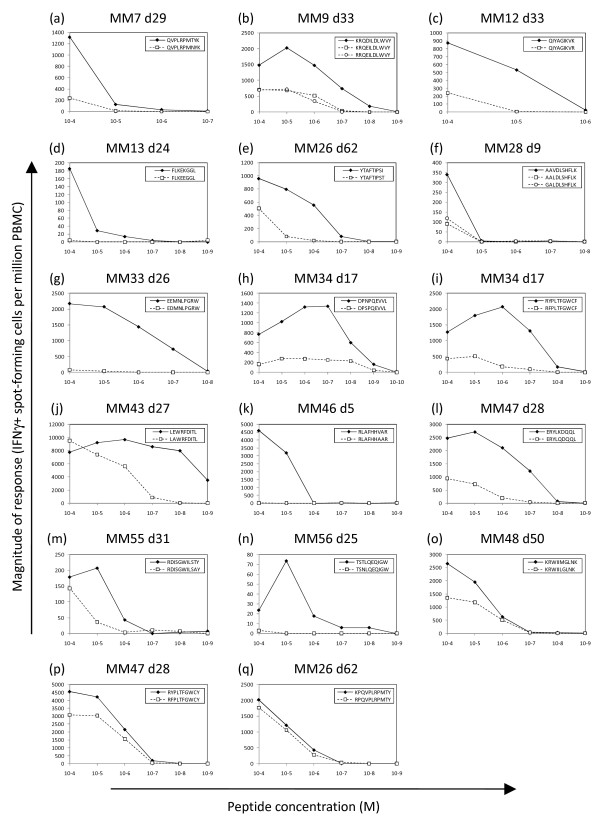
**Comparison of T cell recognition of index sequence and variant sequence peptides in IFNγ ELISPOT assays**. In each patient, PBMC from the specified days following symptom onset were stimulated with log-fold titrations (between 10^-4 ^and 10^-10^M) of index sequence peptide (closed diamonds) and the variant peptide(s) that evolved during the first year post-presentation (open squares and circles). The magnitude of the T cell response (IFNγ spot-forming cells per million PBMC) was measured at each peptide concentration.

For 17/19 of the epitope sequences that underwent intra-epitopic sequence variation during year 1, we had sufficient PBMC to perform IFNγ ELISPOT assays to compare T cell recognition of titrated doses of the index sequence and mutant epitope peptide(s). 14/17 (82%) of the mutant epitope peptides were recognised considerably less well by the primary CD8+ T cell response in the patient where they were selected than the corresponding index sequence peptide, i.e. the half-maximal stimulatory concentration of the mutant peptide was at least 10-fold higher than that of the index peptide (Figure 1, a-n). These were deemed to represent T cell escape variants. In 3/19 cases the mutant peptide(s) were recognised with comparable efficiency to the index sequence peptide and thus failed to meet our criteria for an escape variant (Figure 1, o-q), although the changes may potentially have conferred escape via effects on epitope processing, which we did not address.

These data demonstrate (i) that sequence variation within/adjacent to T cell epitope sequences occurs very commonly during acute/early HIV-1 infection and (ii) that in a minimum of 82% of cases, the mutations evolving within the epitope resulted in impaired recognition by the primary CD8+ T cell response.

### Emergence of T cell responses to escape variant epitopes

**Figure 2:**
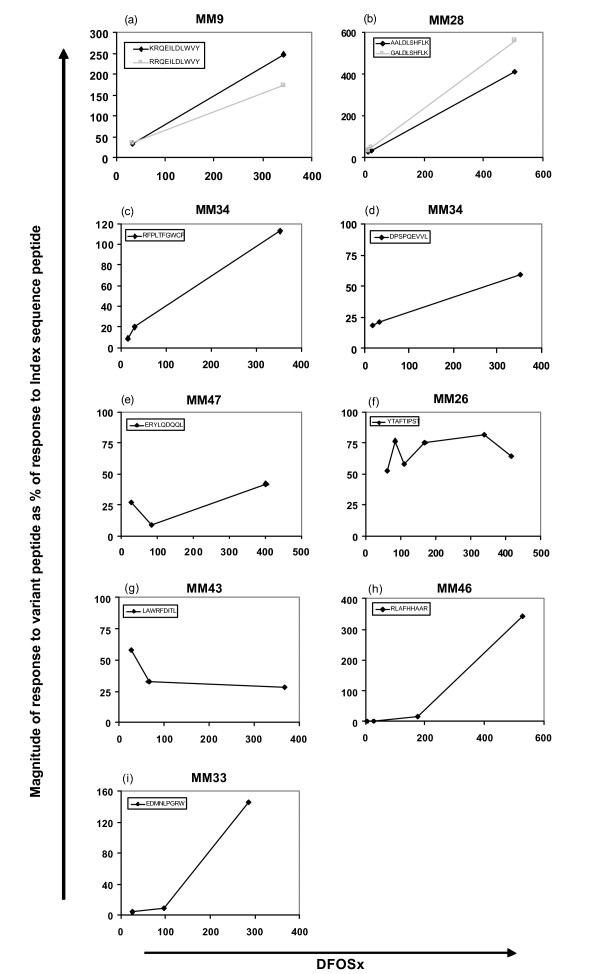
**T cell responses to variant epitope peptides emerge during the first year following presentation with HIV infection**. For 9 epitopes in 8 patients, PBMC from selected time-points during the first year following presentation with HIV infection were titrated against index sequence and variant sequence peptide(s) in an IFNγ ELISPOT assay. The concentration of index sequence peptide stimulating the maximal IFNγ response in the assay was determined. At each longitudinal time-point, the response to this concentration of the variant peptide(s) (black diamonds or grey squares) is shown, expressed as a percentage of the response to the index sequence peptide at the same peptide concentration.

Evidence in the literature shows that new responses to variant epitopes can be mounted during HIV infection [26,27]. As these responses may help to confer continued control of viral replication, we were interested to address whether responses emerged to the variant peptides we defined as escape mutants. For 9/14 of the epitopes where mutations confirmed to confer escape were selected in acute/early infection, we measured T cell recognition of both the index sequence and variant epitope peptides at time-points over the first year of infection by IFNγ ELISPOT assay. A response was considered to have been expanded to the variant peptide(s) if the magnitude of the response to the variant peptide increased over time relative to the response to the index peptide, or if recognition of a previously non-recognised peptide started to be detected. In 7/9 cases, the acute-phase T cell response was capable of at least some recognition of the variant epitope peptide and for 5/7 of these (Figure 2, a-e), the response to the variant increased in magnitude over time relative to the response to the index sequence peptide, consistent with expansion of a response to the variant epitope. In 2/7 cases (Figure 2, f and 2g), although the variant peptide was partially cross-recognised at the earliest time-point, the response to the variant peptide remained relatively stable or reduced relative to the response to the index sequence over time. In 2/9 cases (Figure 2, h and 2i), it appeared that a *de novo *response emerged following evolution of the variant sequence because the acute-phase T cell response showed no recognition of the variant peptide but a response was detected at subsequent time-points. These results suggest that for a subset of HIV-specific CD8+ T cell responses escaped by the virus, the emergence of variant-specific T cell responses over time may allow for a degree of continued control of viral replication.

### The majority of HIV epitope-specific T cell responses maintain stable avidity over the first year of infection

**Figure 3:**
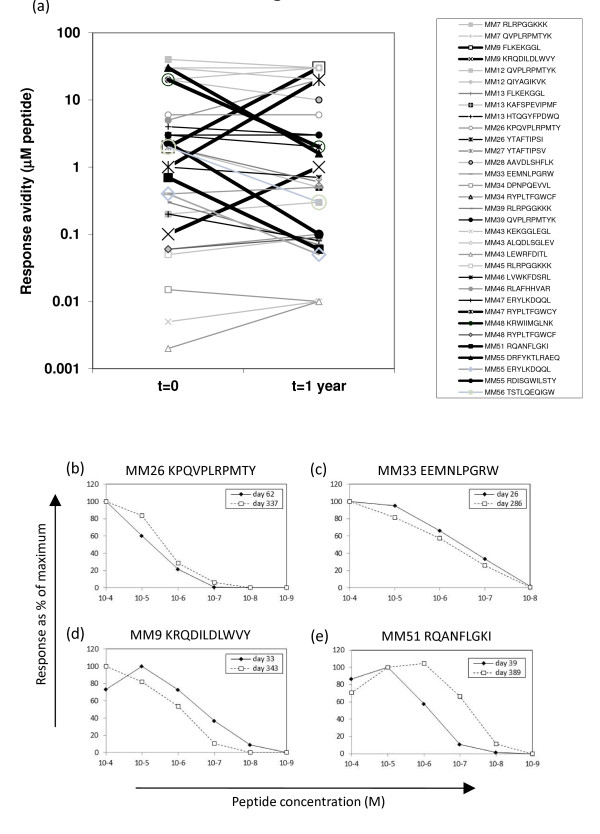
**Comparison of the functional avidity of HIV-specific T cell responses at the earliest sampling time-point tested and ~1 year following symptomatic presentation**. For each of 33 HIV-specific T cell responses, patient PBMC from the earliest sampling time-point available (t = 0), and from t = 1 year following symptomatic presentation with HIV infection were stimulated with log-fold titrations of index sequence peptide (between 10^-4^M and 10^-10^M) in an IFNγ ELISPOT assay. The functional avidity of the response was determined (the peptide concentration stimulating half the maximal IFNγ response in the assay). The graph in (a) shows, for each individual response, the functional avidity at the two time-points. Responses changing in avidity by ≥ 1 log between the two time-points are indicated with bold lines. Examples of data for four representative responses are shown in panels (b-e).

To address whether the avidity of CD8+ T cell responses to the founder virus population was altered over time, we measured the avidity of responses to index sequence epitope peptides at selected time-points over the first year FOSx by peptide-titrated IFNγ ELISPOT assay. All epitope-specific T cell responses studied had avidity values within the μM to nM range at the earliest time-point tested. When the relative avidity of each response at the earliest time-point available (t = 0) was compared to that ~1 year after symptomatic presentation, 7 of 33 responses exhibited a ≥ 10-fold change in avidity over this time. However, of these 7 responses, 4 underwent an increase in avidity; so remarkably only 3/33 (9% of all the responses studied) declined in avidity during this time-frame (Figure 3). As described above, 19/33 (58%) of the responses analysed were directed towards epitopes that underwent intra-epitopic sequence variation within the first year FOSx. When we focused on T cell responses to epitopes that did not undergo intra-epitopic sequence variation within the first year, we found that only 1/14 (7%) showed a ≥ 10-fold decrease in avidity between t = 0 and t = 1 year (and this was a response directed against an epitope where there were changes in the flanking sequence, i.e. none of the 7 responses directed against epitopes in completely invariant sequences declined in avidity over the first year of infection). Reduction in the avidity of HIV-specific T cell responses thus occurs much less frequently than T cell-driven escape during the first year of HIV infection.

## Discussion

It remains unclear why the strong HIV-specific CD8+ T cell responses induced in primary infection are not more effective in controlling virus replication. Mutational escape and reduction in the functional avidity of virus-specific T cell responses represent two mechanisms by which the ability of HIV-specific CD8+ T cells to control viral replication can become impaired. To address which of these may play a more dominant role in reducing CD8+ T cell-mediated control of virus replication in acute/early HIV infection, we measured the relative frequency of sequence variation/escape from, and assessed whether there were alterations in the avidity of 33 epitope-specific CD8+ T cell responses during the first year of HIV infection in a cohort of subjects presenting with symptomatic primary HIV infection, the majority of whom subsequently established moderate-high persisting viral loads.

Amino acid changes were selected for in/around almost 80% of the epitopes studied during the first year of infection. Although these epitopes were derived from different HIV proteins and restricted by different class I alleles, just under half were contained in Nef. This bias likely arose because we deliberately sought to study responses that were mounted during primary infection, and several studies have demonstrated a preferential targeting of acute-phase T cell responses to Nef [3,28]. The extent of the Nef bias may have resulted in our over-estimating the frequency of occurrence of mutational escape because Nef is genetically diverse (reflecting the ability of the virus to tolerate sequence variation in this protein), which is likely to facilitate the evolution of escape mutations. However the Nef bias did not substantially affect the conclusions from our study, as sequence variation was observed in/around 65% of non-Nef epitopes during the first year of infection, and 87% of the intra-epitopic changes in these epitopes were found to confer escape.

Conversely, the approach we used to map the epitopes recognised by the primary HIV-specific T cell response and select a subset of responses for study may have led to under-estimation of the frequency of responses being escaped. Responses were typically mapped at 4-6 months FOSx, then the kinetics of expansion/contraction of responses to optimally-defined epitopes was followed from the earliest available time-point and those which persisted at frequencies high enough for analysis over the first year of infection were selected for study. It is thus conceivable that our mapping may have missed T cell responses that expanded quickly in acute infection, were rapidly escaped, then fell to sub-detectable magnitudes [3,6]; or that we may have excluded responses that were escaped and had declined by 1 year. Rapidly-escaped epitope-specific responses that could have been missed may have included some of the highest avidity responses, as high avidity responses are reported to be preferentially escaped in acute infection [17,29]: this may also have led to under-estimation of the proportion of responses showing a reduction in functional avidity over the first year. Arguing against this however is our detection of a comparable frequency of intra-epitopic sequence variation here and in a recent study of 5 acutely-infected individuals where the HIV-specific T cell response was comprehensively mapped with autologous virus sequence-based peptides and acute/early escape from the entire response was analysed [3].

Of 33 epitopes studied, over half evolved intra-epitopic mutations within the first year following presentation and the majority of these mutations were confirmed to confer T cell escape. There are, however, limitations to the use of this approach for determining the proportion of responses undergoing escape, including the relevance of *in vitro *assays using high peptide concentrations for predicting recognition of viruses expressing the variant sequences [30], and the inability to evaluate the effect of mutations on antigen processing. When analysing epitope-flanking sequences, we found change(s) in regions surrounding 7 epitopes that did not exhibit intra-epitopic variation, which may have affected their processing. The true proportion of T cell responses that was escaped may thus have been higher than we demonstrated. Despite these caveats, we detected a high level of viral sequence variation and escape during the first year of HIV infection. The impact of mutational escape on control of viral replication may however be ameliorated to some extent by the evolution of responses to variant peptides (which we observed in several cases) and/or the fitness costs incurred by the virus in achieving escape, which can be high, particularly for epitopes are located in structurally-conserved proteins such as Gag p24 (reviewed in [31])

When measuring the frequency of alteration in the functional avidity of epitope-specific T cell responses during the first year of HIV infection, we found that < 10% of responses declined in avidity by ≥ 1 log over this period. It is unclear what level of decline in response avidity as assessed in our *in vitro *IFNγ ELISPOT assays would have had a significant impact on *in vivo *control of viral replication, particularly given that the mechanisms by which CD8+ T cells mediate control of HIV replication *in vivo *are not well understood, and the relationship between response avidity and effector capacity may not be the same for all effector functions [24,25]. However, even if a decline in response avidity of ≥ 0.5 log was considered sufficient to have a significant impact on *in vivo *control of viraemia, still only 5/33 (15%) of responses would have been affected by avidity decline over the first year of infection. This was surprising and contrasts with findings made in a previous study by Lichterfeld *et al*. [9], who reported that a large proportion of high avidity T cell responses that were immunodominant in early infection had declined in avidity by chronic infection (typically several years into infection). The methods used to assess response avidity here and in the study by Lichterfeld *et al*. differed, which may have affected the results obtained. Perhaps more importantly, Lichterfeld *et al*. studied only responses that were initially immunodominant and of high avidity, whereas we looked at a cross-section of immunodominant and subdominant responses of both high and low avidities. Work in murine chronic infection models has shown that immunodominant high avidity T cell responses are more likely to become exhausted/deleted in the presence of ongoing antigenic stimulation than initially subdominant responses of lower avidity [32,33]. Further, we assessed changes in response avidity over only the first year of infection: the longer interval between the time-points studied by Lichterfeld *et al*. may have given time for a higher proportion of responses to drop in avidity. Reduction in the avidity of T cell responses may be more common in chronic infection as T cells become exhausted by continued antigenic exposure [34].

It is interesting to consider the mechanisms that may have contributed to the changes in T cell response avidity that we observed. We found one example of a reduction in response avidity in the absence of sequence change in/around the epitope, which may have been due to exhaustion and/or deletion of the highest avidity T cell clones involved in the epitope-specific response. In two other cases, a reduction in functional avidity occurred in association with intra-epitopic sequence variation, hence may have resulted from variant peptide-driven expansion of T cell clones with lower avidity for the index sequence epitope. Interestingly, we also found four examples of increases in response avidity over the first year of infection. Two of these occurred in association with intra-epitopic sequence variation, and in one of these cases it appeared that an escape mutation had been transmitted that then reverted, stimulating expansion of T cells able to recognise the original epitope with higher avidity than the initial response [35]. In the other epitopes, the increases in response avidity may have reflected selection of a subset of higher avidity epitope-specific cells over time [36], or maturation in response avidity in the absence of changes in T cell receptor usage [37].

## Conclusions

The results of this study show that sequence variation and escape occur much more frequently than reduction in the avidity of T cell responses during the first year of HIV infection, suggesting that escape represents a more important means of viral evasion of CD8+ T cell control in acute/early HIV infection (although other mechanisms, such as a decline in the functional capacity of virus-specific CD8+ T cells may also contribute to impairment of T cell control of HIV replication during early infection). The tremendous capacity of the virus to escape from CD8+ T cell responses (shown here and in previous studies) poses a huge problem for the design of HIV vaccines aiming to elicit cell-mediated immune responses, and development of strategies for limiting escape from vaccine-induced T cell responses is paramount. These may include induction of broad T cell responses to multiple viral epitopes, individual components of which are less likely to be escaped [17], targeting conserved epitopes in which viral sequence variation is limited due to structural constraints (reviewed in [31]), and stimulation of T cell responses that can cross-recognise epitope variants efficiently to reduce viral options for escape from T cell control [38,39].

## Methods

### Patients and blood samples

Individuals acutely-infected with HIV-1 were recruited at the Mortimer Market Centre for Sexual Health and HIV Research (London, UK). Subjects were mostly male Caucasians who presented with symptoms of acute retroviral illness. Study approval was obtained from The National Health Service Camden and Islington Community local Research Ethics Committee and blood samples were drawn with written informed consent. Blood was drawn into ethylene diamine tetra-acetic acid (EDTA) (Sigma, Gillingham, UK) and PBMC were isolated over Histopaque 1.077 (Sigma) and cryopreserved. All subjects chose not to receive antiretroviral therapy throughout the study duration with the exception of MM7 who received anti-retroviral therapy from 62-156 DFOSx. The setpoint persisting viral load established in each subject was calculated as described in [40].

### HLA class I typing

Genomic DNA was purified from patient PBMC using a QIAamp DNA blood mini kit (QIAGEN Ltd, Crawley, UK). High resolution class I typing was performed by the Oxford Transplant Centre (Churchill Hospital, Oxford, UK) using a polymerase chain reaction (PCR) method with sequence-specific primer mixes.

### Population sequencing of plasma virus RNA

Population sequencing of plasma viral RNA was performed as previously described [3]. Briefly, HIV-1 RNA was isolated from plasma using a QIAamp viral RNA mini kit (QIAGEN), and cDNA was synthesized from replicate plasma virus RNA preparations using Super-Script III reverse transcriptase (Invitrogen Life Technologies, Carlsbad, CA, USA). Replicate cDNA samples (200-1200 RNA molecules/reaction) were subjected to nested PCR amplification using an Elongase enzyme kit (Invitrogen). All PCR products were sequenced directly. This approach enables detection of variants present at frequencies of 20% or more in the viral quasispecies [41]. Nucleotide changes were considered to be fixed in the viral quasispecies if they evolved to become the sole residue detected over time.

### Synthetic peptides

Peptides were synthesised by FMoc or TBoc chemistry and purchased in a peptide-amino acid format either from Sigma or Mimotopes (Clayton, Australia).

### Mapping of the HIV-specific T cell response

HIV-specific T cell responses were mapped by IFNγ ELISPOT assay using a peptide matrix screening approach as described previously [3]. Briefly, cryopreserved patient PBMC (typically pooled cells from two sample time points within the first 6 months FOSx) were tested by IFNγ ELISPOT assay for responses to matrices of peptides (18-20mers overlapping by 10aa, each present at 10^-5^M) corresponding to the HIV clade B consensus sequence (2001) or (in four patients) the autologous early virus sequence. From the matrix screening a ranked list of potential epitope-containing peptides was deduced, and putative epitope-containing peptides were retested individually at 10^-5^M in a second round assay. Peptides stimulating responses measuring ≥ 3 × background counts and ≥ 50 IFN-γ spot-forming cells per million PBMC were considered positive.

### Measurement of the functional avidity of CD8^+ ^T cell responses

The functional avidity of CD8+ T cell responses at sequential time-points during the first year of HIV infection was determined by peptide-titrated IFNγ ELISPOT assay. Index sequence (earliest autologous sequence determined) peptides were titrated (between 10^-4^M and 10^-11^M) against a constant number of PBMC (1.5-2 × 10^5^/well). The functional avidity was determined as the peptide concentration required to elicit half of the maximal IFNγ response in the assay.

### Analysis of effect of intra-epitopic sequence variation on T cell recognition (escape analysis)

Patient PBMC (1.5-2 × 10^5^/well) were stimulated with log-fold titrations of either index or variant sequence peptide(s) in IFNγ ELISPOT assays. A variant peptide was deemed an escape variant if its half-maximal stimulatory concentration was at least 10-fold higher than that of the index sequence peptide.

## List of abbreviations

DFOSx: days following onset of symptoms; EDTA: ethylene diamine tetra-acetic acid; ELISPOT: enzyme-linked immunosorbent spot; HIV-1: human immunodeficiency virus type 1; HLA: human leukocyte antigen; IFN: interferon; PBMC: peripheral blood mononuclear cells; PCR: polymerase chain reaction; TCR: T cell receptor.

## Competing interests

The authors declare that they have no competing interests.

## Authors' contributions

ELT contributed to the study design, carried out many of the immunological assays and wrote the first draft of the manuscript; JB, SY and XW carried out the viral sequence analysis; KEC carried out some of the immunological assays; MW mapped the HIV-specific T cell responses in some of the patients studied; JT, PP and IW recruited the study subjects, obtained peripheral blood samples from them and provided the clinical and virological data; GMS contributed to the design of the study and oversaw the analysis of the viral sequence data; and PB conceived of, designed and co-ordinated the study, contributed to data analysis and helped to draft the manuscript. All authors read and approved the final manuscript.
